# Management of Incidental Peripheral Pulmonary Arterial Aneurysm by Interventional Radiology

**DOI:** 10.7759/cureus.17337

**Published:** 2021-08-20

**Authors:** Karim Nasra, Neud Kiros, Anthony Diebes, Deep Raole, Matthew Osher

**Affiliations:** 1 Radiology, Ascension Providence/Michigan State University, Southfield, USA; 2 Radiology, Indiana University School of Medicine, Indianapolis, USA; 3 Radiology, Robert Wood Johnson University Hospital, New Brunswick, USA; 4 Interventional Radiology, Ascension Providence/Michigan State University, Southfield, USA

**Keywords:** interventional radiology, embolization, peripheral pulmonary artery aneurysm, pulmonary artery aneurysm, post-infectious pulmonary complications

## Abstract

A peripheral pulmonary arterial aneurysm (PAA) is an abnormal dilatation of the distal pulmonary artery consisting of all three vessel wall layers (the intima, media, and adventitia). It is a rare, potentially life-threatening entity. There is no defined standard for an abnormal amount of dilation of the distal pulmonary vasculature, however, the most common criteria used is a diameter greater than 1.5 times the upper limit of a normal or proximal portion. Despite the rarity of peripheral PAAs, the ability to recognize and diagnose them is important for both radiologists and clinicians. Early recognition is needed because of the high mortality associated with rupture. Consistent guidelines still need to be developed to help clinicians determine when intervention is appropriate. In the interim, endovascular coil embolization has become a mainstay of treatment due to its minimally invasive nature and lower risk of complications when compared to open surgical approaches.

## Introduction

A peripheral pulmonary arterial aneurysm (PAA) is an abnormal dilatation of the distal pulmonary artery consisting of all three vessel wall layers (the intima, media, and adventitia). It is a rare, potentially life-threatening entity. For the main pulmonary trunk, this equates to 43 mm in males and 40 mm in females [1]. There is no defined standard for an abnormal amount of dilation of the distal pulmonary vasculature, however, the most common criteria used is a diameter greater than 1.5 times the upper limit of a normal or proximal portion. Although the true incidence of peripheral PAA is unclear, a study of post-mortem examinations demonstrated an incidence of 8 out of 109,571 (0.007%) [2].

The classification of a PAA begins with anatomic location. A peripheral PAA arises from the distal vasculature, including lobar, segmental, and subsegmental branches [3]. A central PAA is defined as one that arises from a major pulmonary vessel, such as the pulmonary trunk or left/right main pulmonary artery. It is estimated that central PAAs comprise 70% of all PAAs. Additionally, a PAA is classified as a saccular or a fusiform aneurysm. A saccular aneurysm is defined as a non-circumferential outpouching of the vessel wall as opposed to fusiform, which involves the entire circumference.

Presentation is often nonspecific, and many patients are asymptomatic. Those with symptoms may experience some or all of the following: chest pain, dyspnea, hemoptysis, or signs of compression. Superior vena cava (SVC) syndrome is a possible but rare symptom [1]. The diagnosis of a PAA is often found incidentally in imaging, but, in suspected cases, pulmonary angiography is the gold standard as it can differentiate PAA from other vascular malformations, including pulmonary arteriovenous malformation (AVM). CT, MRI, and especially echocardiography are other viable, less invasive alternatives [4].

The most severe and life-threatening complications of PAAs are dissection and rupture. Treatment strategies include endovascular repair, surgical resection, and surgical correction of the underlying congenital disorder. Early recognition is paramount due to the high mortality with rupture, yet there are no established management guidelines [5].

## Case presentation

A 55-year-old man presented to the ED with the main complaint of a severe headache. Additional symptoms included dizziness and nausea. He denied any trauma. He stated that the headache started earlier that day and that headaches were uncommon for him. 

A physical exam of the patient revealed a well-appearing male without neurological deficits. The patient was calm and cooperative. Due to the main presenting symptom of headache, a CT head and a CT angiogram of the head and neck were ordered to look for a potential cerebrovascular cause, such as bleed or aneurysm.

Although no cause of the headache was discovered, there was an incidental discovery of a 1.2 cm nodular opacity in the superior segment of the left lower lobe with features suggestive of a peripheral PAA. Non-emergent CT angiography of the chest was recommended for further evaluation. After treatment with fluids and pain medication, the headache improved, and the patient was discharged home.

**Figure 1:**
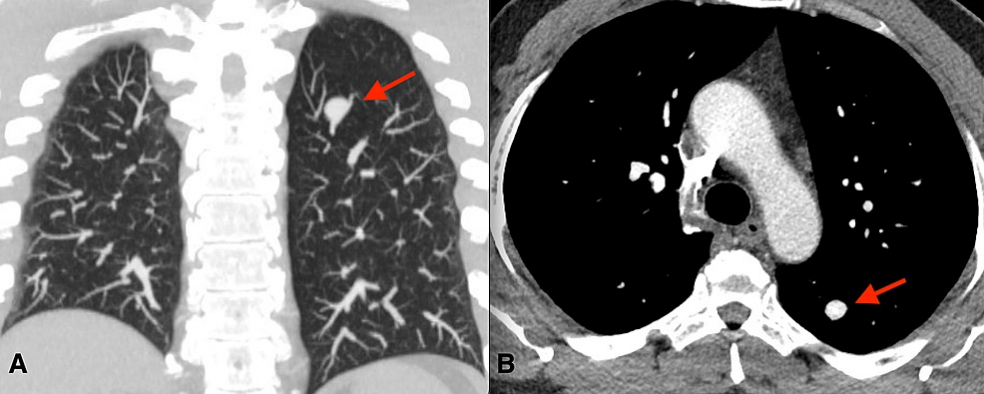
Coronal thin-slab maximum intensity projection (A) and axial soft tissue window through the chest demonstrate an enhancing nodular opacity (red arrow) along the course of the pulmonary artery. No draining vein was identified, and this was compatible with a peripheral pulmonary artery aneurysm.

The patient returned three months later for a CT angiography of the chest. On imaging, a 1.2 x 1.0 x 1.1 cm enhancing structure in the superior segment of the left lower lobe was seen arising from the subsegmental branch of the left lower lobar pulmonary artery, most consistent with a PAA (Figure 1). No draining vein was identified, which would have suggested AVM. Pulmonary angiography was recommended for confirmation and treatment.

**Figure 2:**
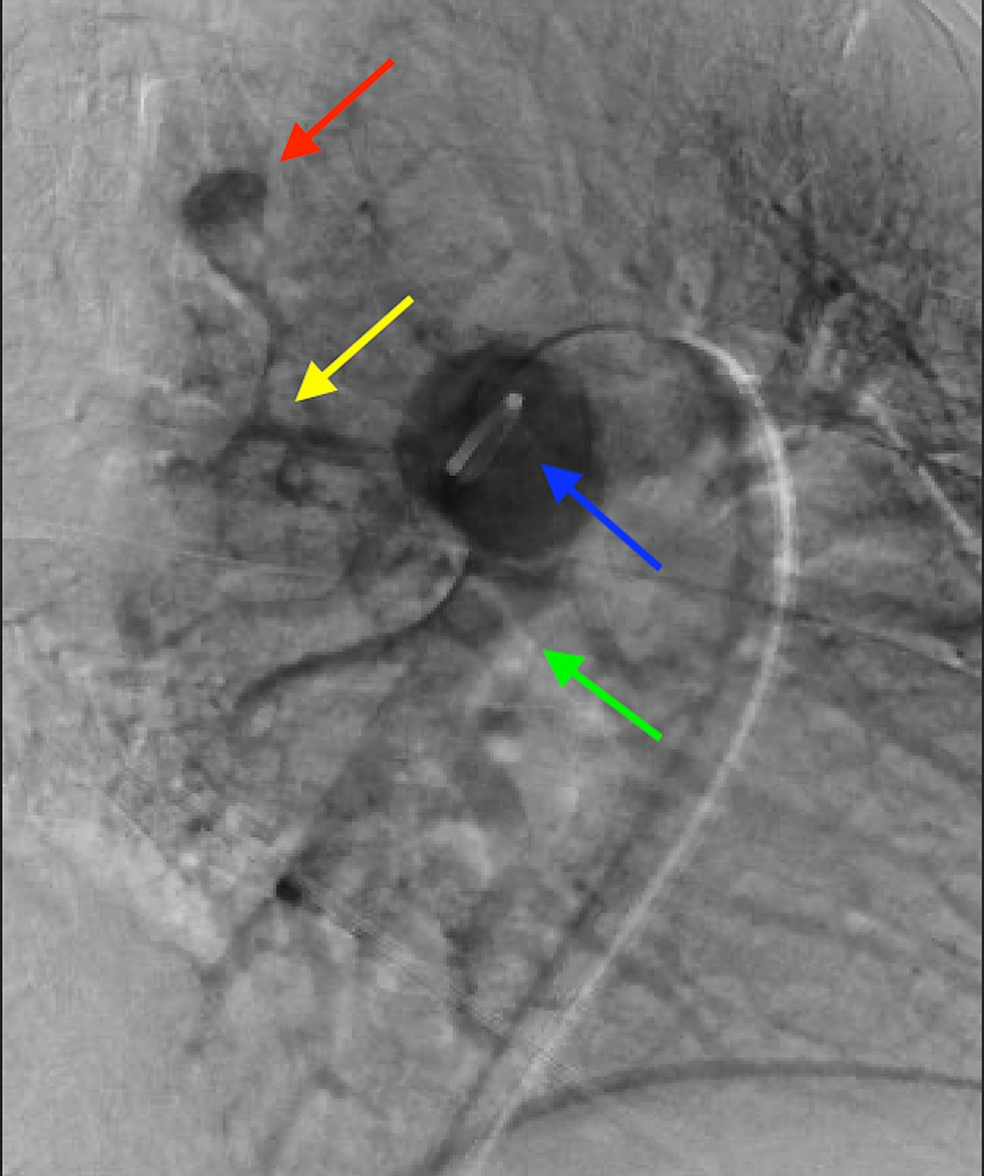
Digital subtraction pulmonary angiography demonstrates the peripheral pulmonary artery aneurysm (red arrow) arising from the superior segmental and subsegmental branches (yellow arrow). A pigtail catheter is seen in the left pulmonary artery (blue arrow). The lower lobe pulmonary artery is also seen (green arrow).

**Figure 3:**
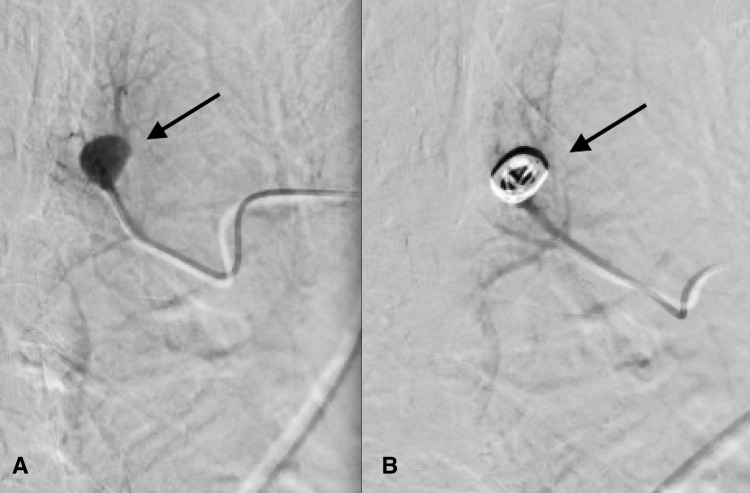
Pre-embolization (A) left subsegmental digital subtraction angiography performed just proximal to the peripheral pulmonary artery aneurysm (black arrow) does not identify a draining vein. Post-embolization angiography (B) demonstrates no significant filling of the aneurysm and preservation of distal blood flow.

The patient returned the following week for intervention. Under ultrasound guidance, access was gained to the right common femoral vein via a four French micropuncture kit and transitioned to an eight French vascular sheath. Using standard guidewire and catheter, a five French pigtail catheter was advanced through the right atrium and ventricle and into the main pulmonary trunk. Pulmonary manometry was performed yielding normal pulmonary arterial pressures. A seven French destination sheath was then advanced into the central main pulmonary artery. Main pulmonary angiography demonstrated normal pulmonary branching anatomy with normal perfusion of the left lung. The left lower lobe aneurysm was visualized. The catheter was advanced into the left pulmonary artery and pulmonary angiography was performed at a steep obliquity to help delineate the anatomy and provide a road map (Figure 2). A selective catheter was then used to select the lower lobar artery, superior subsegmental branch. Gentle hand angiography demonstrating an 11 mm fusiform aneurysm that correlated with the CT findings confirmed no draining vein, proving this to be a true PAA rather than an AVM (Figure 3A).

The catheter was advanced into the aneurysm and coil embolization was performed using a 13 mm x 24 cm detachable coil. Completion angiogram demonstrated no significant residual filling within the aneurysm with maintained perfusion in the peripheral branches (Figure 3B). The catheter was retracted into the main pulmonary artery and manometry revealed no change in pulmonary arterial pressure. All catheters wires were removed and hemostasis was achieved with manual pressure. The patient tolerated the procedure well. After six hours of observation, the patient was discharged home without complication.

## Discussion

Etiologies for PAAs include congenital heart defects, infection, vasculitis, and pulmonary arterial hypertension (PAH) [6]. In a recent review, 25% of reported PAA cases were due to congenital defects, although this number has been reported as high as 50% [1, 6].

The most common congenital heart defects that cause PAAs are patent ductus arteriosus, ventricular septal defect, atrial septal defect, hypoplastic aortic valve, and bicuspid aortic valve [5]. The pathophysiology is likely due to left-to-right intracardiac shunts, the development of Eisenmenger’s syndrome, and associated PAH [5].

In terms of infectious causes, Mycobacterium tuberculosis and Treponema pallidum were historically considered the most common pathogens. However, as a treatment for these bacteria has become more available, Staphylococcus, Streptococcus, and occasionally fungi have become the most common infectious causes of PAAs [7].

An infection can cause an aneurysm due to its ability to erode into the vasculature and cause weakness in the vessel wall [5]. This is the mechanism by which syphilis can cause endarteritis of the vasa vasorum. Septic emboli from patients with bacterial endocarditis can also cause PAAs. This may be seen in patients with a history of IV drug use, immunosuppression, prosthetic heart valve, or congenital heart disease [3].

While a true aneurysm contains all three layers of the vessel wall, a pseudoaneurysm contains only the adventitia, making it more prone to rupture. A Rasmussen aneurysm is a pseudoaneurysm that results from chronic inflammation from a tuberculous cavity. It is reported to occur in 5% of cavitary tuberculosis and has a predilection for the distal vasculature [8]. As a sequela of tuberculosis, it often occurs in the upper lobes.

An important differential diagnosis for a PAA is a pulmonary AVM. This is an abnormal direct connection between the pulmonary arterial and venous circulation that bypasses the capillaries. Due to this bypass of low-resistance vessels, AVMs are high-flow systems. This manifests as an aneurysmal sac present in most AVMs, which can potentially mimic PAA [9]. It is important to distinguish between the two as AVM carries the risk of paradoxical embolism. A key diagnostic difference between the two on angiography is the presence of draining veins in AVM which suggest a connection to the venous circulation.

Treatment strategies for PAAs are varied. Before the advent of endovascular therapy, surgical resection was the only option. Endovascular coil embolization has become a mainstay of treatment as it is minimally invasive and has a lower risk of complication compared to surgical approaches. This is especially true in patients with pulmonary arterial hypertension. A recent review suggested endovascular therapy should be considered as the first-line treatment in all saccular PAAs and in peripheral fusiform PAAs. Surgery is still preferred for central fusiform aneurysms [5]. The risks of endovascular therapy in PAA are largely identical to those of any embolization procedure: site infection/hematoma, contrast-induced nephropathy, and thrombus formation. There have been no studies comparing surgical and endovascular therapy outcomes, likely due to the paucity of PAA cases.

Two questions remain in a diagnosed PAA: How often should we survey these patients and when is treatment appropriate? Accurate guidelines for the intervention of a PAA are needed as there is no significant progress made toward this goal [10]. This is especially relevant in cases where the PAA is enlarging as there isn't a uniform threshold to intervene. Discerning clinical judgment is the best approach we currently have to determine when to intervene. In terms of risk factors, a review by Duijnhouwer AL found high-risk PAAs to be those with pulmonary hypertension in congenital heart disease, rapid PA diameter growth of greater than 2 mm/year, PA pressure greater than 50 mmHg, and tissue weakness due to infection. A standardized set of risk factors may provide a framework on which to create management guidelines, so further epidemiologic studies are warranted to better characterize this rare phenomenon [11].

## Conclusions

Despite the rarity of peripheral pulmonary arterial aneurysms, the ability to recognize and diagnose them is important for both radiologists and clinicians. Early recognition is needed due to the high mortality associated with rupture. Consistent guidelines still need to be developed to help clinicians determine when intervention is appropriate. In the interim, endovascular coil embolization has become a mainstay of treatment due to its minimally invasive nature and lower risk of complications when compared to open surgical approaches.
